# Analysis of the impact of a diabetes education program on glycemic control and prevalence of chronic complications

**DOI:** 10.20945/2359-3997000000541

**Published:** 2022-12-01

**Authors:** Amanda Oliveira S. Monteiro Silveira, Mabel Duarte Alves Gomides, Geraldo Sadoyama

**Affiliations:** 1 Universidade Federal de Catalão Faculdade de Medicina Catalão GO Brasil Faculdade de Medicina, Universidade Federal de Catalão, Catalão, GO, Brasil; Associação dos Diabéticos do Sudeste de Goiás, Catalão, GO, Brasil; Associação dos Diabéticos do Sudeste de Goiás Catalão GO Brasil; 2 Universidade Federal de Catalão Faculdade de Medicina Catalão GO Brasil Faculdade de Medicina, Universidade Federal de Catalão, Catalão, GO, Brasil; 3 Universidade Federal de Catalão Instituto de Biotecnologia Catalão GO Brasil Instituto de Biotecnologia, Universidade Federal de Catalão, Catalão, GO, Brasil

**Keywords:** Diabetes mellitus, diabetes complications, health education, multidisciplinary research

## Abstract

**Objective:**

Diabetes mellitus (DM) is a chronic disease of increasing importance in public health, associated with chronic complications including retinopathy, neuropathy, and kidney, cardiovascular and cerebrovascular disease. This study assessed the impact of strategic DM education actions on glycemic control and prevalence of chronic complications in patients with DM.

**Subjects and methods:**

Retrospective, quantitative, cohort study at a diabetes patients association comprised of a multidisciplinary team. In all, 533 individuals with DM were included. Sociodemographic and clinical data were collected using questionnaire and medical records. Of these, 333 patients evaluated for 12 to 24 months, with type 2 DM (T2DM, n = 317) and other types of DM (n = 16), were selected to collect data on retinopathy and diabetes kidney disease (DKD).

**Results:**

There was a predominance of elderly individuals, low education level, women, high rate of overweight and obesity, physical inactivity, dietary errors, dyslipidemia, and T2DM. More patients with T2DM versus type 1 DM had optimal glycemic control (46.3% vs. 12.2%, respectively; p < 0.001). The impact of the educational processes was demonstrated by the analysis of the initial and final glycated hemoglobin (HBA1c) levels. There was an increased prevalence of individuals with well-controlled DM during follow-up (prevalence ratio [PR] 2.76, 95%, p = 0.001), along with a significant reduction in retinopathy (PR: 0.679, p = 0.001) and albuminuria (PR: 0.637, 95%, p = 0.002) when these variables were evaluated in well-controlled versus uncontrolled HbA1c groups.

**Conclusions:**

A multidisciplinary approach with integration and quality was associated with improvements in DM control and reduced occurrence of chronic DM complications.

## INTRODUCTION

Diabetes mellitus (DM) is a chronic disease with an insidious and progressive course. The importance of DM in public health has been growing due to increasing incidence, prevalence, morbidity, mortality, and economic costs associated with the disease (1). According to 2021 data from the International Diabetes Federation (IDF), there are currently around 537 million people with diabetes worldwide (10.5% of the world population), and this projection is estimated to increase to 783 million in 2045 (2). It is important to note that about half of these individuals are unaware of having the disease and are part of a group of patients with a higher risk of chronic complications associated with diabetes, *i.e.*, retinopathy, neuropathy, and kidney, cardiovascular and cerebrovascular disease (2,3).

Previous studies have demonstrated the importance of adequate and sustained glycemic control in patients with type 1 DM (T1DM) and type 2 DM (T2DM) in reducing chronic complications, emphasizing the central role of maintaining glycated hemoglobin (HBA1c) levels within values considered ideal (<7.0%) in order to motivate healthcare professionals and patients to achieve adequate and continuous control of blood glucose levels (4,5).

The education strategy of the Diabetes Self-Management Education and Support (DSMES) is a fundamental tool for successful treatment of the disease. The DSMES comprises actions developed to motivate patients to modify their lifestyle habits and adopt self-care behaviors. In this strategy, basic information and training on DM are administered to patients and their families and caregivers so that they assimilate the knowledge and techniques and develop skills and attitudes to manage DM, thus improving the patients’ quality of life and preventing and/or delaying the occurrence of chronic diabetes complications (6–8).

Thus, centers that care for individuals with DM should implement the DSMES recommendations in a multidisciplinary way, to be delivered by teams comprised of professionals in several healthcare areas, including endocrinology, nephrology, cardiology, ophthalmology, nutrition, nursing, dentistry, physical education, and psychology (6,9).

Based on these considerations, the objective of this study was to evaluate the impact on glycemic control and on the prevalence of chronic complications of diabetes of strategic educational actions carried out by the multidisciplinary team of the Associação dos Diabéticos do Sudeste de Goiás (ADISGO). The study also sought to identify the sociodemographic and clinical characteristics of the population served at ADISGO, as well as to observe the outcomes of the educational programs offered by the institution.

The importance of this study would be to promote improvements in the provision of services offered, aiming at greater adherence to treatment and reduction of complications related to the disease.

## SUBJECTS AND METHODS

Retrospective, quantitative, cohort study of applied nature carried out at ADISGO, a non-profit institution with the purpose of providing free services to individuals with DM in the population of Catalão and southeast Goiás. ADISGO is composed of 3,700 associate members (patients with DM) and a team of professionals that includes specialists in cardiology, endocrinology, ophthalmology, nutrition, nursing, podiatry, and psychology, among others. The protocol of the study was approved by the research ethics committee of the Federal University of Goiás (protocol number 1.963.714).

ADISGO promotes strategic actions of DM guidance and education through assistance to patients with DM by a multidisciplinary team: at least 2 consultations a year with an endocrinologist, cardiologist, nutritionist, and 1 annual consultation with an ophthalmologist; individualized care for patients and family members, by a nurse, regarding the use of devices necessary for treatment (handling of blood glucose monitors, insulin application with syringes, pens, and infusion pumps); promotion of monthly scientific meetings at the institution's headquarters with topics related to DM and associated complications, being necessary to attend at least nine meetings out of a total of 12 per year, in order to have access to consultations. Member's participation in meetings is recorded by means of an electronic card; campaign for detection and prevention of DM complications, guidelines for the prevention of diabetic foot through feet examination during medical consultation, podiatric care and courses annual on carbohydrate counting and dietary reeducation, and experimental diet cooking services.

The study population included all associate members enrolled at ADISGO. Of these, we selected those patients who participated in consultations offered by the multidisciplinary team (n = 694). After evaluating the medical records of these patients, we excluded those whose last appointment had taken place more than 24 months before, had a change in place of residence, who no longer attended the institution, and those who had deceased before the data collection, resulting in a final sample of 533 patients. These were grouped according to the diagnosis of T1DM, T2DM, or other types of diabetes (OTD). The OTD category included patients with DM who did not fulfill the diagnostic criteria for T1DM or T2DM and included cases of DM secondary to diseases of the exocrine pancreas, related to the use of medications such as glucocorticoids, and other forms of DM eventually linked to monogenetic factors but without etiological elucidation. From the entire cohort of patients (n = 533), we collected sociodemographic and clinical data using a questionnaire and information retrieved from medical records, including information on the patients’ adherence to the dietary plan recommended by the ADISGO nutritionist and family history of DM in first-degree relatives.

Next, we selected those patients (n = 333), frequent consultations in the last 12 to 24 months, whose medical records showed results of two to three HbA1c tests measured using high-performance liquid chromatography (HPLC), three albuminuria tests (represented in mg of albumin per g of creatinine), fundoscopy, and creatinine and/or glomerular filtration rate estimated by the Chronic Kidney Disease Epidemiology Collaboration (CKD-EPI) equation. We excluded patients with T1DM from the collection of data about chronic complications since about half of these patients had less than 5 years of disease duration. Since the evaluation on diabetes kidney disease (DKD) and fundoscopy are recommended to be done preferably after this time point, collection of these data would not have been possible.

From the group of patients (n = 333), with T2DM (n = 317) and OTD (n = 16) who used the services offered by the multidisciplinary team at ADISGO and the DM educational services, we evaluated the initial HbA1c level (*i.e.*, the HbA1c requested at the first consultation at ADISGO). Then, we evaluated the final HbA1c level (*i.e.*, the HbA1c from the last consultation at the institution). The mean time between the initial and final HbA1c was 3.4 ± 2.0 years. We considered HbA1c < 7% as “well controlled” and ≥ 7% as “uncontrolled.” We also collected data on chronic DM complications (retinopathy and DKD).

### Statistical analysis

The statistical analyses were performed using the Statistical Package for Social Sciences (SPSS) for Windows, version 23.0 (IBM Corp., Armonk, NY, USA) (10). The descriptive analysis is presented using mean values. We also used tables with absolute and relative frequencies of the collected data. The Shapiro-Wilk test was used to analyze the normal distribution of the quantitative variables. Inferential analysis was performed using the chi-square, Fisher's, and McNemar's tests for comparison between percentage values, and the Student's *t* test and analysis of variance (ANOVA) with Tukey's *post hoc* for comparison of quantitative variables.

A significance level of 5% was adopted in all analyses. The strength of the association between each of the qualitative explanatory variables and response variables was analyzed by calculating the prevalence ratio (PR) followed by the respective 95% confidence intervals (95% CIs). For quantitative variables, the Glass delta (difference magnitude), 95% CI, and the common language effect size (CLES) were calculated.

## RESULTS

### Clinical and sociodemographic characteristics

Among the 533 patients analyzed in the study, there was a predominance of the female sex (65.5%), low educational level (56.5% had incomplete elementary education and 9.4% were illiterate), T2DM (87.6%) over T1DM (7.7%) and OTD (4.7%), and age group of 45-79 years (82%).

The body mass index (BMI) and waist circumference (WC) of the participants are shown in Table 1. Among patients with T1DM aged ≤ 18 years, BMI was normal in 91.7% of them and in the overweight range in 8.3% of them. In patients with T2DM, the BMI indicated overweight in 37.7% and obesity in 45.3% (3.9% of these had class III obesity).

**Table 1:** Body mass index and waist circumference in the study patients according to the type of diabetes (ADISGO 2017)

Type of diabetes	Body mass index (kg/m²)		Waist circumference (cm)	
	≤18 years** Mean (min-max)	>18 years*** Mean (min-max)	≤18 years** Mean (min-max)	>18 years*** Mean (min-max)
T1DM (n = 41)*	18.22 (14.3-22.0)	24.25 (18.0-31.4)^a^	67.3 (53.0-84.0)	86.92 (80.0-107.0)^a^
T2DM (n = 467)	N/A	29.95 (17.5-53.3)^b^	N/A	101.40 (75.0-150.0)^b^
OTD (n = 25)	N/A	29.51 (17.1-57.0)^b^	N/A	97.70 (77.0-137.0)^b^

* ≤18 years, n = 24; > 18 years, n = 17.

** One sample Student's *t* test (p = 0.001).

*** One-way analysis of variance (ANOVA).

a and b , significant difference, Tukey's test (p < 0.05).

min: minimum value; max: maximum value; T1DM: type 1 diabetes mellitus; T2DM: type 2 diabetes mellitus; OTD: other types of diabetes; N/A: not applicable.

Regarding the WC, 79.2% of the patients aged ≤ 18 years in the T1DM group had WC values (adjusted for sex and age) below the 90th percentile (*i.e.*, normal; p = 0.001).

An evaluation by sex of mean BMI in the T2DM group showed a higher BMI in the female (30.4 ± 5.9 kg/m²) compared with the male (29.0 ± 4.3 kg/m²) sex.

Since patients with T2DM and OTD had very similar clinical characteristics, we grouped both for the analysis of chronic complications.

An adequate glycemic control (HbA1c < 7.0%) was observed in 12.2% of the patients with T1DM and in 46.3% of those with T2DM. In the analysis by stratified HbA1c values, patients with T1DM were less likely to fit in the range ≤ 6.9% than patients with T2DM (p = 0.001; PR = 0.18; 95% CI: 0.072-0.452). As the HbA1c value increased progressively, there was an increase in the patient's risk of presenting an HbA1c level between 9% and 11.9%, a finding that was significant in the T1DM group (Table 2).

**Table 2:** Stratification by range of glycated hemoglobin according to the type of diabetes (n = 508) (ADISGO 2017)

Glycated hemoglobin (HPLC)	T1DM (n = 41)		T2DM (n = 467)		p	PR	95% confidence interval
	N	%	N	%			
≤6.9%	5	12.2	216	46.3	<0.001	0.180	(0.072-0.452)
7-7.9%	8	19.5	120	25.7	0.456	0.719	(0.341-1.517)
8-8.9%	6	14.6	54	11.6	0.611	1.280	(0.562-2.915)
9-9.9%	9	22	39	8.4	0.009	2.695	(1.369-5.305)
10-10.9%	8	19.5	24	5.1	0.002	3.606	(1.818-7.149)
11-11.9%	3	7.3	5	1.1	0.020	4.934	(1.917-12.699)
≥12%	2	4.9	9	1.9	0.220	2.317	(0.638-8.411)

Fisher's exact test. HPLC: high-performance liquid chromatography; T1DM: type 1 diabetes mellitus; T2DM: type 2 diabetes mellitus; n: number of cases; %: percentage of cases; p: p value; PR: prevalence risk.

### Relationship of the impact of strategic actions on diabetes guidance and education developed by a multidisciplinary team on the glycemic control of patients with T2DM e OTD receiving care at ADISGO

As shown in Table 3, of the 333 individuals evaluated, 120 had a well-controlled initial HbA1c (value < 7%), while 151 had a well-controlled final HbA1c (chance of an initially well-controlled individual remaining well controlled after the follow-up and interventions delivered by the multidisciplinary ADISGO team = 2.76, 95% CI 2.18-3.51, p = 0.001). Of the 213 patients with uncontrolled DM according to the level of HbA1c, 182 remained poorly controlled in the final evaluation.

**Table 3:** Relationship of the impact of strategic actions on diabetes education using the final and initial glycated hemoglobin indicator (n = 333) (ADISGO, 2017)

		Final glycated hemoglobin		p	PR	95% CI	Total
		Well controlled	Uncontrolled				
Initial glycated hemoglobin	Well controlled	92	28	0.001	2.76	2.18-3.51	120
	Uncontrolled	59	154				213
Total		151	182				333

McNemar test. p: p value; PR: prevalence ratio; 95% CI: 95% confidence interval.

### Relationship of glycated hemoglobin values with sociodemographic, clinical, and chronic complications in patients T2DM e OTD

Table 4 shows the final HbA1c level between the groups of well-controlled and uncontrolled patients in relation to the sociodemographic and clinical variables at ADISGO.

**Table 4:** Relationship of final glycated hemoglobin with sociodemographic and clinical variables (ADISGO, 2017)

Variables	HbA1c < 7%	HbA1c ≥ 7%	p	Δ (CI min-max);	CLES
	(mean ± SD)	(mean ± SD)			
Age (years) (n = 333)	60.91 ± 10.04	60.29/10.32	0.581	0.06 (0.16-0.28)	51.7
BMI (kg/m^2^) (n = 333)	29.05 ± 4.91	30.72 ± 5.89	0.006	0.28 (0.09-0.53)	58.7
WC (cm) (n = 322)	99.65 ± 10.36	102.12 ± 11.80	0.05	0.21 (0.01-0.44)	56.3
DM duration (months) (n = 331)	102.14 ± 70.1	144.34 ± 70.18	<0.001	0.60 (0.38-0.82)	66.5

Student's *t* test. HbA1c: glycated hemoglobin; SD: standard deviation; BMI: body mass index; WC: waist circumference; DM: diabetes mellitus; p: p value; Δ (CI min-max): Glass delta (minimum-maximum confidence interval); CLES: common language effect size.

Regarding the variable “dietary plan,” the group with well-controlled HbA1c had significantly greater odds (almost twice more) of following the recommended dietary plan compared with the group with uncontrolled HbA1c (Table 5). Patients in the group with well-controlled HbA1c were 3.6 times more likely to be using hypoglycemic agents (p < 0.001). For the variable “family history,” p was also significant. The remaining variables in Table 5 showed no significant differences.

**Table 5:** Relationship between final glycated hemoglobin and clinical variables, ADISGO 2017

		HbA1c < 7%		HbA1c ≥ 7%		p	PR	95% CI
		Yes	No	Yes	No			
Dietary plan (n = 321)		106	39	68	108	0.000	1.880	1.525-2.318
Physical exercise (n = 310)		37	100	38	135	0.303	1.134	0.884-1.454
Medications in use (n = 330)*								
	Hypoglycemic agent	127		76		<0.001	3.611	2.434-5.358
	Insulin	1		10		0.014	0.195	0.030-1.274
	Hypoglycemic agent and insulin	21		95		<0.001	0.302	0.202-0.452
Smoking (n = 333)		11	140	11	171	0.650	1.100	0.715-1.690
Alcoholism (n = 333)		4	147	10	172	0.198	0.755	0.534-1.067
Hypertension (n = 333)		96	55	133	49	0.063	0.811	0.644-1.022
Dyslipidemia (n = 333)		110	39	149	33	0.078	0.797	0.607-1.046
Stroke (n = 332)		9	141	6	176	0.238	1.388	0.741-2.600
Family history (n = 330)		75	75	116	64	0.008	0.758	0.613-0.938

Chi-square test. HbA1c: glycated hemoglobin; n: number of cases; p: p value; PR: prevalence ratio; 95% CI: 95% confidence interval.

* For the medications in use, yes/no was not applied.

The chronic complications of diabetic retinopathy and DKD were analyzed in relation to well-controlled versus uncontrolled final HbA1c. As shown in Table 6, the risks of these complications reduced significantly with the educational intervention.

**Table 6:** Relationship of final glycated hemoglobin and chronic complications variables (ADISGO 2017)

Variables		HbA1c < 7%		HbA1c ≥ 7%		p	PR	95% CI
		Yes n (%)	No n (%)	Yes n (%)	No n (%)			
Retinopathy (n = 313)		20 (13.9)	124 (86.1)	51 (30.0)	118 (70.0)	0.001	0.679	0.559-0.825
Diabetes kidney disease								
	Increased UAE* (n = 315)	6 (4.16)	138 (95.8)	25 (14.6)	146 (85.4)	0.002	0.637	0.519-0.783
	Decreased GFR** (n = 321)	29 (20.0)	116 (80.0)	40 (22.7)	136 (77.3)	0.554	0.931	0.739-1.173

Chi-square test. HbA1c: glycated hemoglobin; n: number of cases; %: percentage of cases; p: p value; PR: prevalence ratio; 95% CI: 95% confidence interval; UAE: urinary albumin excretion;

* ≥30 mg albumin/g creatinine; GFR: glomerular filtration rate;

** <60 mL/min/1.73 m².

## DISCUSSION

Among the factors analyzed, we observed that the prevalence of DM increased with aging up to the age of 79 years, with a predominance above the age of 60 years. As already documented in several other studies, this occurrence is attributed to changes in the aging process, as well as reduced physical activity and unhealthy changes in dietary habits (11,12).

When we evaluated the prevalence of DM by sex, we observed a considerable predominance of the female over the male sex. This profile has already been observed in other studies and occurs due to a greater demand for healthcare services among women and, therefore, a greater opportunity for medical diagnoses (12). However, several other factors have been identified to justify the increased frequency of DM in women. A study has observed that women with polycystic ovary syndrome (PCOS) have a four-time greater risk of developing T2DM at an early age compared with women without PCOS (13). Another important aspect is weight gain between pregnancies, which increases the risk of gestational DM (14) and, in turn, increases the risk of T2DM development 3 to 6 years after delivery (2).

Changes in lifestyle are considered fundamental in DM care and are part of the education for DM self-care (15). The current increased prevalence of obesity is noteworthy, and the disease is considered one of the main risk factors for the development of T2DM in addition to contributing to increased risk of hypertension and dyslipidemia, which in turn increase the risk of cardiovascular events. In this sense, we observed high rates of overweight and obesity in the T2DM group (close to 38% and 45%, respectively).

Data from the Surveillance of Risk and Protective Factors for Chronic Diseases by Telephone Survey (VIGITEL), released by the Ministry of Health (16), revealed that 55.7% of the respondents were overweight and 19.8% were obese, with documented increases over the last 12 years of 30% and 67% in the prevalence of overweight and obesity, respectively. A progressively increased prevalence of obesity in different age groups has been observed worldwide since the 1970s (17).

When we evaluated the WC and BMI values in relation to the sex of the participants, we observed that female compared with male participants presented WC values that were more distant from the desirable range and higher BMI. A study evaluating the prevalence of obesity from 2013 to 2014 in the United States observed that 35.0% of the men and 40.4% of the women fit into this category, and showed a linear trend towards obesity in the study period (18).

Thus, recognizing the occurrence of overweight and obesity in this population is necessary to encourage lifestyle changes, contributing to improvements in treatment and reducing the risk of complications. For many overweight or obese patients, the literature describes that a weight loss above 5% is necessary to produce beneficial results in controlling blood glucose, lipids, and blood pressure (19).

The difference between the T1DM and T2DM groups in terms of HbA1c levels – the primary outcome variable in the present study – was significant. An adequate glycemic control was observed in 12.2% and 46.3% of the patients with T1DM and T2DM, respectively. In a multicenter Brazilian study with 6,671 adults with DM, only 10.4% and 26.8% of the patients with T1DM and T2DM, respectively, were within glycemic targets considered adequate (20). In another cross-sectional Brazilian study with 5,750 patients with T1DM receiving care covered by the Unified Health System, only 26% of the patients had well-controlled DM (21).

A North American study with 10 years of follow-up has shown that almost 50% of the individuals with DM do not reach and maintain the target HbA1c level of < 7% (22). In that study, only 14.3% of the patients were within the ideal goals of HbA1c levels, blood pressure, low-density lipoprotein (LDL)-cholesterol, and smoking cessation (22).

The results of the present study are in line with data from the literature that show that patients with T1DM present glycemic decompensation more frequently than patients with T2DM. This observation is explained, in part, by difficulties in obtaining supplies and devices required to treat the disease, social and cultural difficulties in handling these devices, and psychological challenges in coping with the disease. In addition, the lack of knowledge about the disease on the part of caregivers and family members, and also from the patients themselves, associated with inadequate training and integration techniques among healthcare professionals make treatment adherence even more difficult (1,23,24).

ADISGO offers monthly DM education meetings, an opportunity to promote increased adherence to DM treatment. However, not all ADISGO members attend these meetings, often due to lack of motivation, especially among patients with T1DM. Thus, it is important to plan education strategies for patients in different age groups and social and cultural levels to bring the patient closer to the institution and the educational processes to achieve the goals established by the patient and the multidisciplinary team (23,25).

Of note, patients with T1DM require more immediate attention and specific proposals for better adherence to treatment. Especially for children and adolescents, the main objective should guide the independence and autonomy necessary for each age group, always aiming at self-management of blood glucose, insulin application, and nutritional management according to daily activities (1,25). In order for the information to reach specific populations in certain age groups, several educational and technological resources have been used, such as educational games, online or in-person lectures, and the creation of websites and applications for cell phones and social networks. ADISGO has addressed these processes, but further improvement and continuous training of the team is necessary for better use of these tools (26).

It is important to understand that the educational process must occur continuously in the relationship between professionals, patients, family members, and caregivers. For this reason, joint working is fundamental for the integration of the participants. The combined work of each professional within their diversities is essential, providing integrated diagnostic, educational, and therapeutic intervention so that each professional evaluates the conditions and needs of each patient and can discuss the required management with other professionals (27,28).

A systematic review has shown a significant 0.6% reduction in HbA1c levels with education strategies; this reduction is comparable to that obtained with medications, albeit without the side effects (29). As described in the UKPDS 38 (30), each 0.9% reduction in HbA1c is associated with reductions of 25%, 10%, and 6% in the occurrence of microvascular complications, mortality from DM, and mortality from all causes, respectively.

The evaluation of the impact of multidisciplinary services offered by the ADISGO professionals team was carried out by comparing the initial and final HbA1c levels of each patient. The result of this analysis demonstrated, with statistical significance, almost three times greater odds for well-compensated patients to remain within the goals of glycemic control after the educational intervention by the ADISGO team and a reduction in the number of poorly controlled patients in the final evaluation. Therefore, coinciding with data from the literature, the present study reinforces the importance of a reference center for individuals with DM, with a multidisciplinary team capable of collaborating in the control and maintenance of the patients’ glycemic levels (1,7).

Also regarding the analysis of the primary outcome (HbA1c), we observed a relationship between sociodemographic, clinical, and chronic complications. The group with uncontrolled HbA1c showed significantly higher values of BMI and WC and longer DM duration compared with the group with well-controlled HbA1c. This, in turn, showed greater discipline in adhering to the dietary plan, less family history of DM, and a greater number of patients using oral hypoglycemic agents.

When the data on chronic complications were evaluated in relation to the group with well-controlled and uncontrolled HbA1c for the variable UAE in the context of DKD, a significant reduction in the risk of its occurrence was observed for the group of well-controlled patients, although the same did not occur for the variable GFR < 60 mL/min/1.73 m^2^. Several studies have demonstrated the beneficial effect of glycemic control on the progression of DKD. In the UKPDS 33 study with patients with newly diagnosed T2DM, there was a 30% reduction in the development and progression of DKD, an effect that persisted for 10 years (5). The Diabetes Control and Complications Trial (DCCT) also found an association between hyperglycemia and microvascular injury, including DKD, and risk reduction with good control (31).

In addition to the association with hyperglycemia, the risk of DKD increases with the duration of the DM and association with other risk factors that are modifiable (hypertension, dyslipidemia, and smoking) and non-modifiable (age, non-white ethnicity, and genetic predisposition) (32). For this reason, multifactorial intervention by a multidisciplinary team is essential to prevent DKD and its progression (33), as well as changes in lifestyle, recommendations for the appropriate and safe use of drugs are required, with constant monitoring and an individual approach to increase adherence to self-care practices (34).

There was a significant reduction in the prevalence of retinopathy in the well-controlled compared with the uncontrolled HbA1c group. This observation confirms that poor glycemic control is an important factor in the progression of retinopathy, while maintaining glycemic control within goals decreases its risk (4,5).

Other factors identified as increasing the risk of developing retinopathy include DKD, hypertension, and dyslipidemia. Thus, intervention on various DM complications is essential to address this complication better (30,33,35).

In conclusion, the strategic actions of DM education and guidance promoted by the multidisciplinary ADISGO team improved the glycemic control of the patients with T2DM e OTD, reflected by greater odds of the patients maintaining good glycemic control, and significantly reduced the prevalence of chronic DM complications such as retinopathy and DKD. The recognition of the sociodemographic and clinical characteristics of the assisted population at ADISGO should be valued as a strategic tool for planning future educational interventions, so that these proposed actions meet the institution's desires and demands. For educational DM processes to be incorporated more widely into healthcare services, universal and integrated involvement between patients, family members, caregivers, society, professionals, and healthcare managers is essential.
